# The impact of gut microbiota manipulation with antibiotics on colon tumorigenesis in a murine model

**DOI:** 10.1371/journal.pone.0226907

**Published:** 2019-12-20

**Authors:** Jae Gon Lee, Chang Soo Eun, Su Vin Jo, A-reum Lee, Chan Hyuk Park, Dong Soo Han

**Affiliations:** Department of Internal Medicine, Hanyang University Guri Hospital, Hanyang University College of Medicine, Guri, Korea; Toho University Graduate School of Medicine, JAPAN

## Abstract

It has been suggested that manipulation of gut microbiota using antibiotics can inhibit colitis-associated colorectal cancer (CAC) in a mouse model. We investigated whether timing of gut microbial manipulation using antibiotics affects colon tumorigenesis in the azoxymethane (AOM)/dextran sodium sulfate (DSS)-induced CAC model. CAC was induced in C57BL/6 mice by injection of 12.5 mg/kg AOM followed by three rounds of 1.7% DSS exposure. There were six groups based on timing of antibiotic administration. Colonic inflammation, proliferation, and tumorigenesis were evaluated after animal sacrifice. High-throughput sequencing of the mice feces was performed to characterize changes in gut microbiota. Full-time antibiotic treatment significantly decreased the number and size of tumors, histological scores, and expression of pro-inflammatory cytokines compared to the AOM/DSS group without antibiotic treatment. The early and late antibiotic groups, antibiotic administration from the first and second rounds of DSS to the end of the study, showed significantly lower histological scores and tumor burden. In contrast, the pretreatment antibiotic group, antibiotic administration from 3 weeks prior to AOM to the first round of DSS, did not exhibit decreased tumorigenesis. Principal coordinate analysis showed similar gut microbial community structures among the full-time, early, and late antibiotic groups, whereas other groups showed distinct gut microbial profiles. There was a positive correlation between number of tumors and number of operational taxonomic units. Colonic tumorigenesis was attenuated by antibiotic administration, except for that only prior to DSS administration, suggesting that gut microbial changes should be maintained throughout the entire period of inflammation to suppress tumorigenesis.

## Introduction

Inflammatory bowel disease (IBD), characterized by chronic relapsing inflammation of the gastrointestinal tract, has emerged as a substantial public health challenge worldwide [1]. IBD causes debilitating gastrointestinal symptoms and can also cause colitis-associated cancer (CAC) over the long term [2]. In patients with IBD, the cumulative incidence of CAC is up to 20% in ulcerative colitis (UC) and 8% in Crohn’s disease (CD) [3]. The duration and severity of IBD are associated with development of CAC [3].

Although the pathogeneses of IBD and CAC are not completely understood, it is generally accepted that excessive immune responses to gut microbiota and subsequent chronic inflammation are involved [4]. Unlike sporadic CRC, mutations in the p53 gene are highly abundant at an early stage of dysplasia during development of CAC. In addition, intestinal barrier dysfunction promotes bacterial invasion, sustains intestinal inflammation, and eventually results in mutations in the adenomatous polyposis coli (APC) gene and carcinoma development [5]. Several specific microbes, including *Escherichia coli*, *Bacteroides fragilis*, and *Fusobacterium nucleatum*, have been reported to be associated with colonic tumorigenesis; however, a single causative organism has not yet been identified [6].

There are trillions of commensal bacteria in the human gut, composed predominantly of species from *Firmicutes*, *Bacteroidetes*, and *Proteobacteria* [7], and a growing body of evidence suggests that changes in composition of the microbial community as a whole may contribute to colonic tumorigenesis [3]. Previous animal studies have reported that gut microbial manipulation with antibiotic treatment may inhibit development of CAC [8, 9]. However, since several factors are involved in the sequential stages of CAC, it is important to assess the effect of gut microbial manipulation on development of CAC during different time frames. Therefore, we investigated whether timing of gut microbial manipulation through antibiotic administration affects colon tumorigenesis in the azoxymethane (AOM)/dextran sodium sulfate (DSS)-induced murine CAC model.

## Materials and methods

### Mice

C57BL/6 mice (female, 6-week-old) were obtained from Orient Bio (Seongnam, Korea) and maintained under specific pathogen-free conditions in an accredited animal facility at Hanyang University. The mice were co-housed in groups with 23 ± 3°C temperature, 50 ± 20% humidity, a 12/12-hour light/dark cycle, and free access to food and water. All mice were fed with standard mouse chow (LabDiet 5053, Orient Bio, Korea). To minimize animal suffering and determine the humane endpoints, mice were monitored daily for the following signs of distress: weight change, hair loss, abnormal eye opening, reduced physical activity, and abnormal posture. The criteria for determining the humane endpoint are shown in S1 Table. All experiment procedures were performed according to the guidelines outlined and approved by the Animal Experimental Ethics Committee of Hanyang University (approval number: 2017-0126A).

### Induction of colitis-associated tumorigenesis

The mice received a single intraperitoneal injection of 12.5 mg/kg AOM. On the fifth day of AOM injection, the mice were fed water that contained 1.7% DSS for five days, followed by untreated water feeding for 14 days. This process was repeated two more times, resulting in a total of three rounds of DSS administration. The control group was given phosphate buffered saline intraperitoneally and normal drinking water without DSS. Mice were euthanized by isoflurane inhalation 14 days after the last round of DSS administration.

### Antibiotic treatment

**Fig 1:**
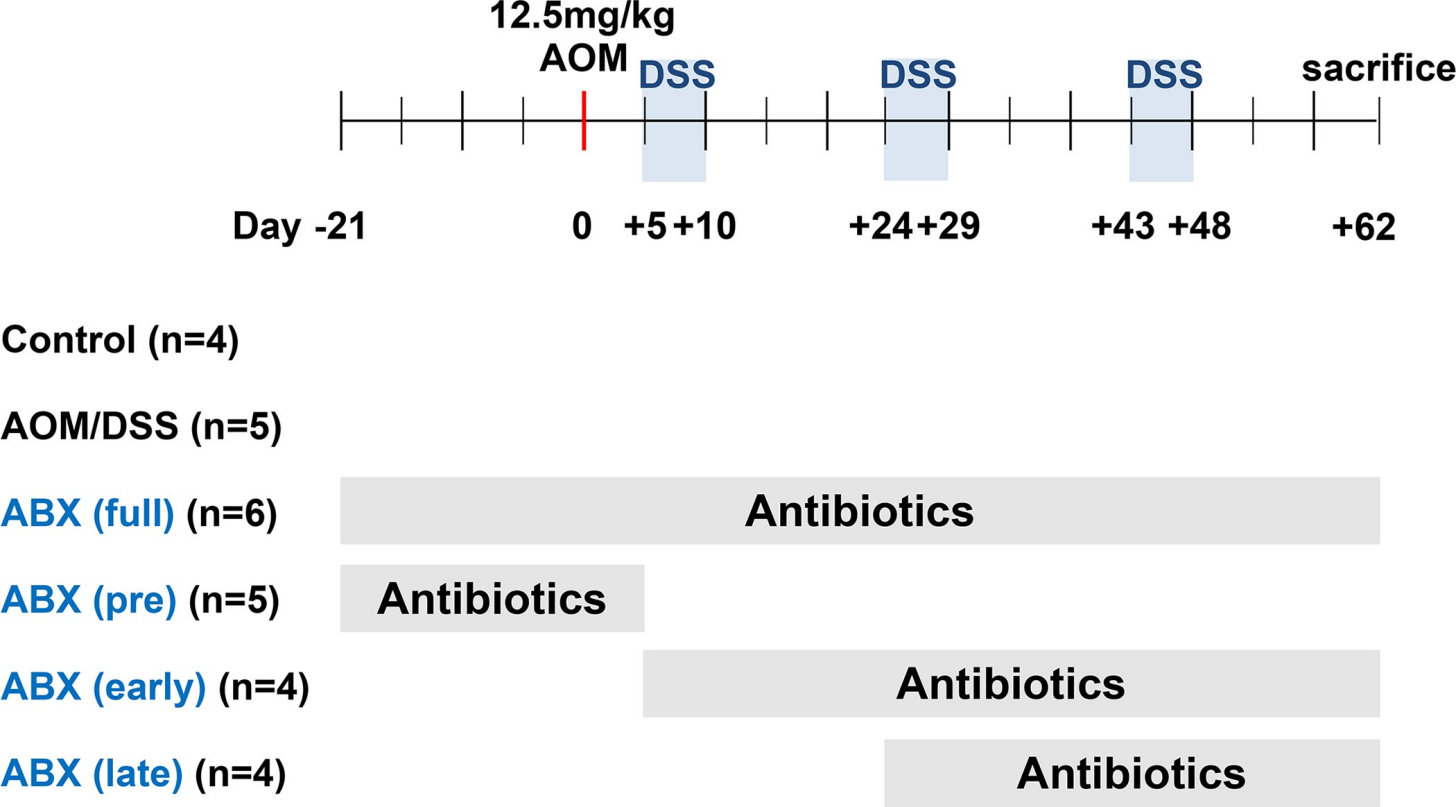
Study protocol. Antibiotics cocktails consisted of ampicillin, neomycin, metronidazole, and vancomycin. AOM, azoxymethane; DSS, dextran sodium sulfate; ABX, antibiotics.

Antibiotic cocktails that contained ampicillin (500 mg/L), neomycin (1 g/L), metronidazole (1 g/L), and vancomycin (250 mg/L) were administered in drinking water. The mice were divided into 6 groups of antibiotic administration timings: (1) control group (n = 4), no AOM/DSS and no antibiotics; (2) AOM/DSS group (n = 6), AOM/DSS without antibiotics; (3) full-time antibiotic group (n = 6), antibiotic administration from 3 weeks prior to AOM to the end of the study; (4) pretreatment antibiotic group (n = 6), antibiotic administration from 3 weeks prior to AOM to the beginning of the first round of DSS; (5) early antibiotic group (n = 4), antibiotic administration from the first round of DSS to the end of the study; (6) late antibiotic group (n = 4), antibiotic administration from the second round of DSS to the end of the study. One mouse in the AOM/DSS group and one in the pretreatment group were euthanized because they met the humane endpoint before the end of the experiment. No mice were found dead during the experiment. Finally, 4 mice in the control group, 5 in the AOM/DSS group, 6 in the full-time antibiotic group, 5 in the pretreatment antibiotic group, 4 in the early antibiotic group, and 4 in the late antibiotic group completed the experiment and were analyzed. The study protocol is shown in Fig 1.

### Gross and histological assessments

After euthanasia, colons were extracted to measure colon length and number of tumors and to determine histological scores. Digital photographs were captured, and number of tumors in the entire colon was documented. The colon tissues were fixed in 4% paraformaldehyde, embedded in paraffin, and stained with hematoxylin and eosin for histological assessments. Three of the authors, who were blinded to the slide information, measured the histological scores of the distal colon by summing the following scores used in a previous study: degree of inflammation (0–4), epithelial defects (0–4), crypt atrophy (0–4), degree of dysplasia/neoplasia (0–4), and extent of dysplasia/neoplasia (0–4) (Table 1) [10].

**Table 1:** Histological grading system.

Criteria		Score
Inflammation	Normal	0
	Small leukocyte aggregates in mucosa and/or submucosa	1
	Coalescing mucosal and/or submucosal inflammation	2
	Coalescing mucosal inflammation with prominent multifocal submucosal extension +/- follicle formation	3
	Severe diffuse inflammation of mucosa, submucosa, and deeper layers	4
Epithelial defects	None	0
	Focally dilated glands and/or attenuated surface epithelium, decreased goblet cells	1
	Focally extensive gland dilation and/or surface epithelial attenuation	2
	Erosions (mucosal necrosis terminating above muscularis mucosae)	3
	Ulceration (full-thickness mucosal necrosis extending into submucosa or deeper)	4
Crypt atrophy (in region most affected)	None	0
	<25%	1
	25–50%	2
	50–75%	3
	>75%	4
Dysplasia/neoplasia	Normal	0
	Aberrant crypt foci, dysplasia characterized by epithelial cell pleomorphism, plump & attenuated forms, gland malformation with splitting, branching, and infolding	1
	Polyploid hyperplasia/dysplasia, moderate dysplasia characterized by pleomorphism, early cellular & nuclear atypia, piling & infolding, occasional cystic dilation, bulging towards muscularis mucosae & projection into lumen, loss of normal glandular, mucous, or goblet cells	2
	Adenomatous and/or sessile hyperplasia/dysplasia; gastrointestinal intraepithelial neoplasia or carcinoma in situ, marked dysplasia confined to mucosa, features as above but greater severity, frequent & sometimes bizarre mitoses	3
	Intramucosal carcinoma (extension of severely dysplastic regions into muscularis mucosae)	3.5
	Invasive carcinoma: Submucosal invasion (differentiate from herniation) or any demonstrated invasion into blood or lymphatic vessels, regional nodes, or other metastasis	4
Area of dysplasia/neoplasia	None	0
	<10% surface area	1
	10–25% surface area	2
	25–50% surface area	3
	>50% surface area	4

### Reverse transcription PCR (RT-PCR)

Total RNA was extracted from colon tissues using Hybrid-R Total RNA Isolation Kit (GeneAll Biotechnology, Seoul, Korea) and quantified using a Biospec-nano spectrophotometer (Life Science, Columbia, MD, USA). RT-PCR was performed 30 times at 95°C for 2 min, 95°C for 30 sec, 55–65°C for 30 sec by each primer, 72°C for 1 min, and 72°C for 5 min. The PCR products were resolved by electrophoresis on 1.5% agarose gels that contained Safe-Pinky DNA gel staining solution (GenDEPOT), and bands were visualized using a ChemiDoc XRS+ System (Bio-Rad, CA, USA). The primer sequences used for PCR are shown in Table 2.

**Table 2:** Primers used in the study.

Target gene	Primer	Sequence (5’ to 3’)	Annealing temperature
β2m	Forward	TGACCGGCTTGTATGCTATC	55°C
	Reverse	CAGTGTGAGCCAGGATATAG	
IL-10	Forward	GTCATCGATTTCTCCCCTGTG	60°C
	Reverse	CCTTGTAGACACCTTGGTCTTGG	
TNF-α	Forward	GCCTCTTCTCATTCCTGCTTG	60°C
	Reverse	CTGATGAGAGGGAGGCCATT	
IL-1B	Forward	GGAGAACCAAGCAACGACAAAATA	55°C
	Reverse	TGGGGAACTCTGCAGACTCAAAC	
IL-6	Forward	ATGGATGCTACCAAACTGGAT	60°C
	Reverse	TGAAGGACTCTGGCTTTGTCT	
IFN-γ	Forward	CTTCCTCATGGCTGTTTCTGG	55°C
	Reverse	ACGCTTATGTTGTTGCTGATGG	
IL-17A	Forward	ATCCCTCAAAGCTCAGCGTGTC	55°C
	Reverse	GGGTCTTCATTGCGGTGGAGAG	
IL-22	Forward	TTGAGGTGTCCAACTTCCAGCA	55°C
	Reverse	AGCCGGACGTCTGTGTTGTTA	
IL-18	Forward	ACTGTACAACCGCAGTAATACGC	60°C
	Reverse	AGTGAACATTACAGATTTATCCC	

### DNA extraction and 16S rRNA gene sequencing

Mouse fecal samples were collected before antibiotic administration and before the mice were sacrificed and were immediately stored at -80°C. To extract bacterial DNA, bacterial walls were pulverized using the phenol/chloroform extraction and bead beating method, and the purified DNA was extracted using PowerFecal DNA Isolation Kit (MOBIO Laboratories, USA).

To amplify the extracted DNA, primers for the V3-V4 region of the 16S rRNA gene were used as follows: forward, TCGTCGGCAGCGTCAGATGTGTATAAGAGACAGCCTACGGGNGGCWGCAG; reverse, GTCTCGTGGGCTCGGAGATGTGTATAAGAGACAGGACTACHVGGGTATCTAATCC. Gene amplification conditions were initial denaturation at 95°C for 5 min, denaturation 35 times at 95°C for 40 sec, primer annealing at 57°C for 40 sec, extension at 72°C for 60 sec, and final elongation at 72°C for 60 sec. Amplified 16S rRNA PCR products were quantified, purified, and sequenced using the Miseq platform (Illumina). The short or extra-long reads in the sequences were trimmed, and the filtered sequence was classified using CD-HIT-DUP. Chimeric reads were identified, and small noise sequences were removed. Sequences with 97% accordance among the remaining representative readings were classified as operational taxonomic units (OTUs). We then assigned a taxonomy for each OTU representative sequence using the QIIME pipeline. Alpha diversity of each group was compared with Chao 1 richness index. In addition, to determine the total bacterial load in the gut, quantitative 16S rRNA PCR on stool samples was performed. The real-time PCR was performed by the 7500 Real-Time PCR System (Applied Biosystems, Foster City, CA, USA) in a 2-step procedure using TOPreal qPCR 2X PreMIX (enzynomics, Daejeon, Republic of Korea). The following primers were used: forward, GTGSTGCAYGGYTGTCGTCA; reverse, ACGTCRTCCMCACCTTCCTC. All reactions were performed in a 96-well plate using the following cycling conditions: 40 cycles of 95°C for 30 sec, 63°C for 30 sec, and 68°C 1 min. Using the Ct (ΔΔCt) method, the value of each control sample was set at 1 and used to calculate the fold-change of target genes. Principal coordinate analysis was performed to evaluate the structural changes of the gut microbial community. The complete genome sequence data have been deposited in the NCBI Sequence Read Archive under BioProject accession number PRJNA560633.

### Statistical analysis

Continuous and categorical variables were compared among groups using the Mann-Whitney test and the chi-square or Fisher’s exact tests, respectively. A *p* value <0.05 was considered statistically significant. All statistical procedures were conducted using IBM SPSS Statistics 20.0 (IBM Corp., Armonk, NY, USA) and R (version 3.5.2; R Foundation for Statistical Computing, Vienna, Austria).

## Results

### Tumorigenesis was regulated differently according to the timing of antibiotic administration

**Fig 2:**
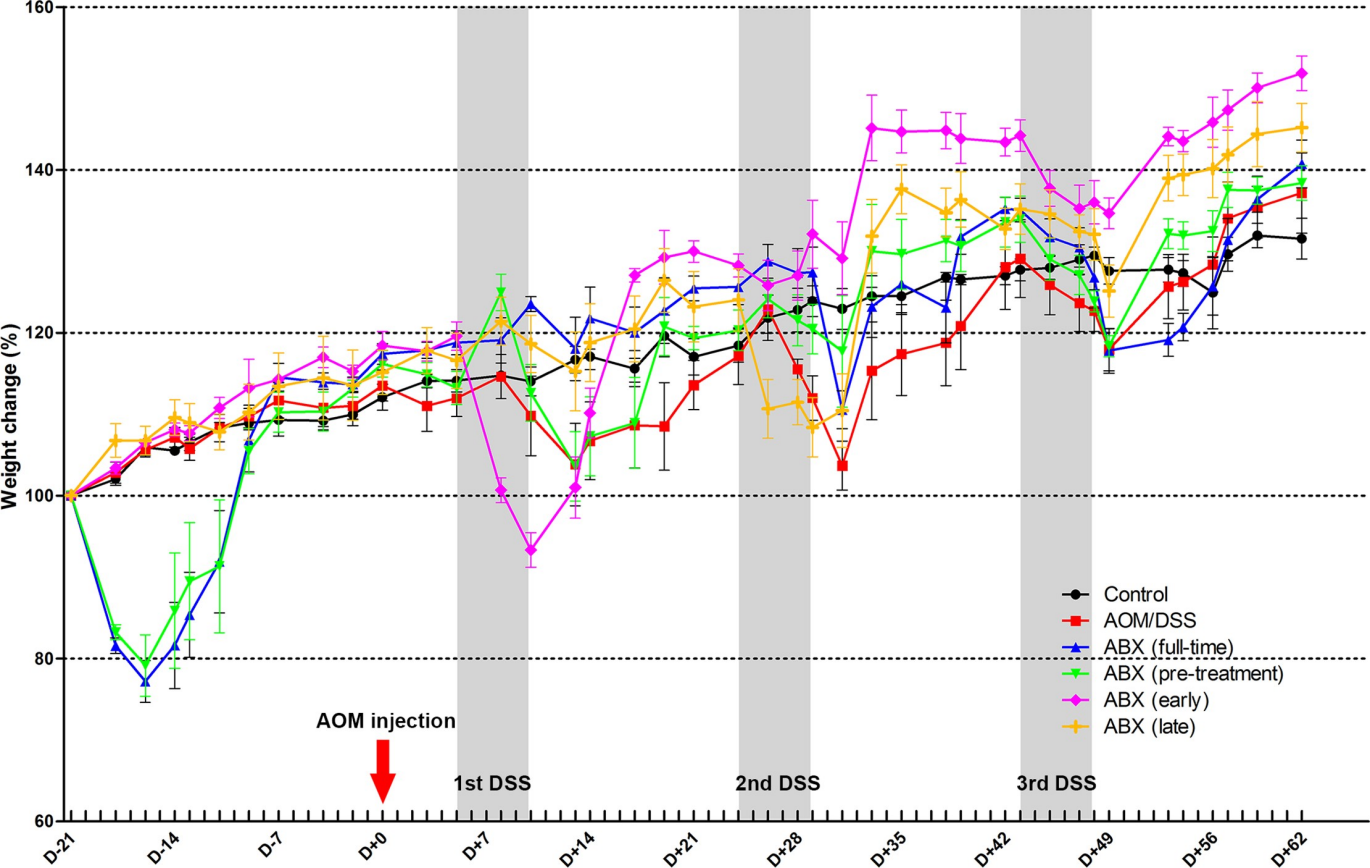
Weight change in each group during the study period. Body weight was expressed as percentage change over initial body weight. Azoxymethane was injected on day 0. Gray shades represent each round of DSS administration. Body weight decreased at the onset of antibiotic treatment and during DSS administration. AOM, azoxymethane; DSS, dextran sodium sulfate; ABX, antibiotics.

The overall weight of the mice in the study decreased at the onset of antibiotic treatment and during DSS administration. Weight loss due to the antibiotic treatment gradually recovered over time. At the end of the study, there was no significant difference in body weight between groups (Fig 2).

**Fig 3:**
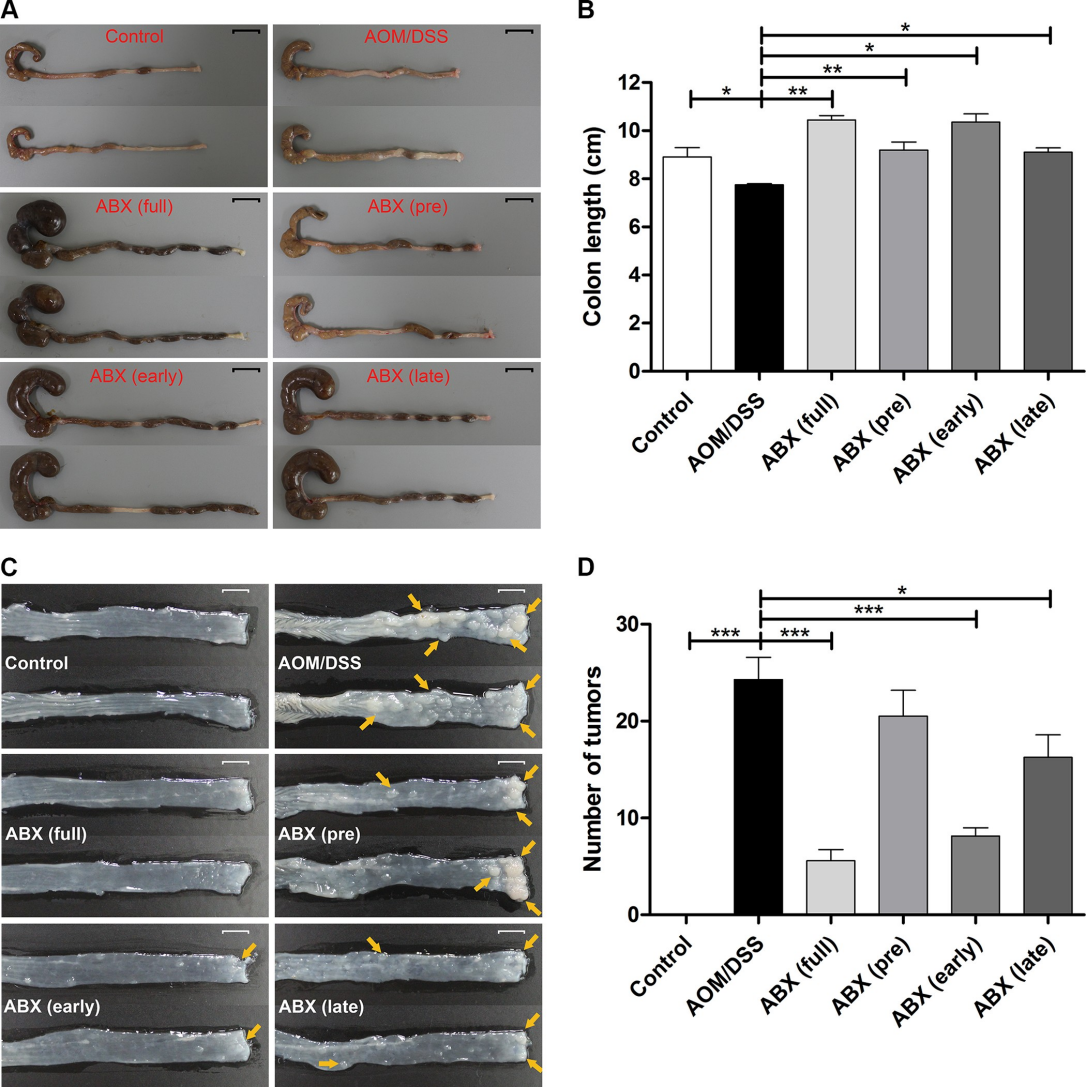
Colon tumorigenesis was attenuated differently depending on the timing of antibiotic administration. (A) Representative images of the harvested colons. Scale bar, 1cm. (B) Colonic length was significantly shorter in the AOM/DSS group than in the control group. (C) Representative images of colonic tumors. Scale bar, 1cm. (D) Compared with the AOM/DSS group, the number of tumors in the entire colon was significantly reduced in the full-time, early and late antibiotic groups. However, there was no significant decrease in number of tumors in the pretreatment group than the AOM/DSS group. AOM, azoxymethane; DSS, dextran sodium sulfate; ABX, antibiotics. * *p*<0.05, ** *p*<0.01, *** *p*<0.001.

Fig 3 shows macroscopic findings from the extracted colons among groups. Colonic length was significantly shorter in the AOM/DSS group than in the control group (mean colon length: 7.76 cm vs. 8.90 cm, *p* = 0.016). All groups that received antibiotic treatments had longer colonic lengths compared with the AOM/DSS group (mean colon length: full-time antibiotic group, 10.45 cm; pretreatment antibiotic group, 9.2 cm; early antibiotic group, 10.35 cm; late antibiotic group, 9.1 cm).

The number of tumors was significantly lower in the full-time, early antibiotic, and late antibiotic groups than in the AOM/DSS group (mean number of tumors: 5.6, 8.1, and 16.3 vs. 24.3, respectively, *p*<0.001). The decrease in number of tumors was more prominent in the full-time antibiotic group than in the late antibiotic group (5.6 vs. 16.3, *p* = 0.004). In contrast, there was no significant difference in number of tumors between the pretreatment antibiotic group and the AOM/DSS group (20.5 vs. 24.3, *p* = 0.302) (Fig 3C and 3D).

These results indicate that the degree of suppression of tumorigenesis varies according to the timing of microbial manipulation with antibiotics.

### Colonic inflammation was alleviated by antibiotic treatment and positively correlated with tumor burden

**Fig 4:**
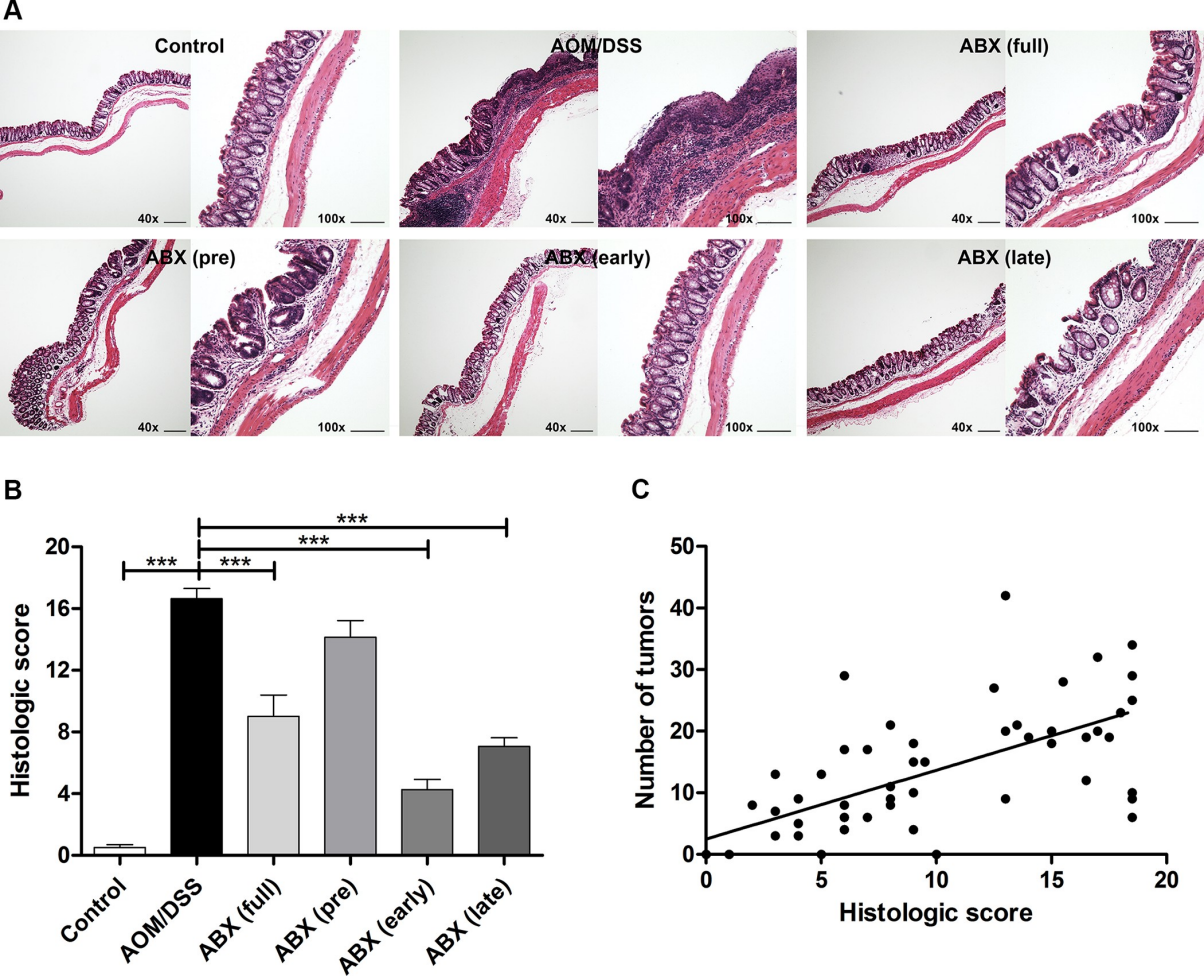
Administration of antibiotics alleviated colonic inflammation, which was associated with suppressed tumorigenesis. (A) Representative histological findings at the end of the experiment. Images were taken from the distal part of the colon at 40x and 100x magnifications. Scale bar, 200 μm. (B) Histologic scores of the distal colon were significantly lower in the full-time, early, and late antibiotic groups than in the AOM/DSS group. However, histological scores were not significantly decreased in the pretreatment group. (C) Histologic score and number of tumors were positively correlated. AOM, azoxymethane; DSS, dextran sodium sulfate; ABX, antibiotics. *** *p*<0.001.

Fig 4 shows histological findings of the extracted colons. The histologic scores of the full-time, early, and late antibiotic groups were significantly lower than that of the AOM/DSS group (9.0, 4.3, and 7.1 vs. 16.7, respectively, *p*<0.001). However, there was no significant difference in histologic score between the pretreatment antibiotic group and the AOM/DSS group (14.2 vs. 16.7, *p* = 0.149). Contrary to the results of the most marked reduction of tumor burden in the full-time antibiotic group, the alleviation of colonic inflammation was most pronounced in the early antibiotic group. Overall, however, the histologic scores and number of tumors were positively correlated (Pearson correlation coefficient R^2^ = 0.585).

These results demonstrate that the alleviation of colonic inflammation also varies with the timing of antibiotic administration, and there was a positive correlation between colonic inflammation and tumor burden.

### Cytokine expression varied with the timing of antibiotic treatment

**Fig 5:**
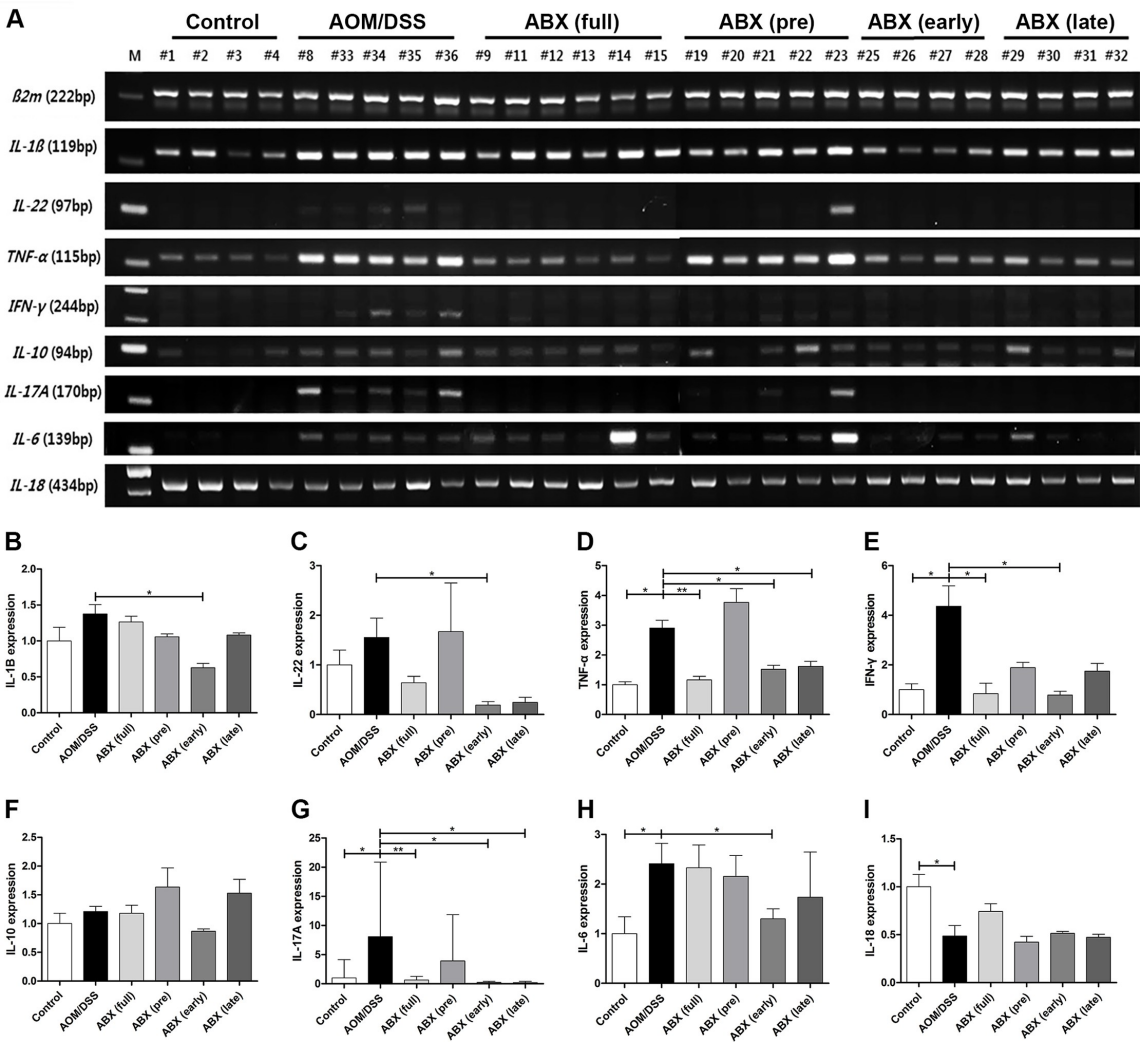
Cytokine expression varied according to the timing of antibiotic treatment. RNA was extracted from colon tissue harvested at the end of the experiment. Semi-quantitative analyses of cytokine expression demonstrate that the antibiotic-treated groups had significant alterations in expression of various cytokines compared with the AOM/DSS group. Size markers for PCR analysis were indicated in 100 base pair units. Full-time, early, and late antibiotic groups showed significant reductions in expression of (D) TNF-α and (G) IL-17A compared with the AOM/DSS group. The expression of (E) IFN-γ was significantly decreased in the full-time and early antibiotic groups, but not in the late antibiotic group. However, expression of (B) IL-1β, (C) IL-22, and (H) IL-6 was significantly reduced in the early antibiotic group but not in the full-time antibiotic group. There was no significant difference in expression of any cytokines in the pretreatment group compared to the AOM/DSS group. AOM, azoxymethane; DSS, dextran sodium sulfate; ABX, antibiotics. * *p*<0.05, ** *p*<0.01.

The mRNA expression profiles of TNF-α, IFN-γ, IL-17A, and IL-6 were increased and higher in the AOM/DSS group than in the control group. Antibiotic treatment decreased mRNA expression of proinflammatory cytokines in the colonic tissue explants. The full-time, early, and late antibiotic groups showed significant reductions in mRNA expression of TNF-α and IL-17A compared with the AOM/DSS group. The expression of IFN-γ was significantly decreased in the full-time and early antibiotic groups, but not in the late antibiotic group. On the other hand, expression of IL-1β, IL-22, and IL-6 was significantly decreased in the early antibiotic group but not in the full-time antibiotic group. In addition, there was no significant difference in mRNA expression of any cytokines between the pretreatment antibiotic group and the AOM/DSS group (Fig 5).

These results demonstrate that the antibiotic administration reduce the expression of pro-inflammatory cytokines such as TNF-α, IFN-γ, and Th17 cytokines. The expression of cytokines differed according to the timing of antibiotic administration.

### Antibiotic treatment significantly altered gut microbial composition

**Fig 6:**
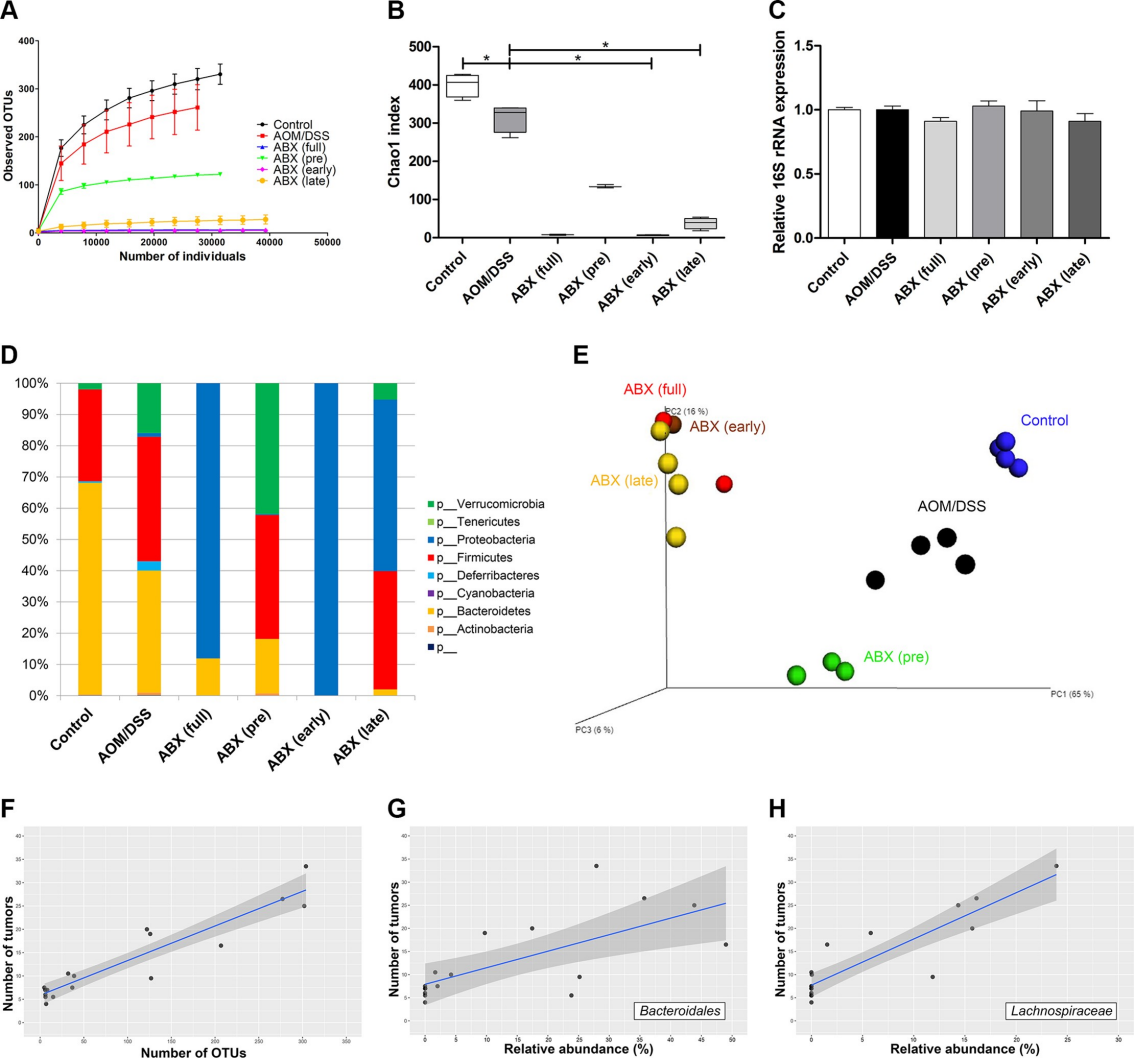
Antibiotic treatment significantly altered gut microbial composition. (A) Chao 1 indices were significantly lower in the antibiotic-treated groups than in the control, especially in the early and late antibiotic groups. (B) Rarefaction curves showed a significantly reduced number of operational taxonomic units in the full-time, early, and late antibiotic groups. (C) Quantitative 16S rRNA PCR revealed no significant difference in bacterial load between the control, AOM/DSS, and antibiotic-treated groups. (D) At the phylum level, the relative abundance of Bacteroidetes was decreased, while that of Proteobacteria was increased in the antibiotic-treated groups. (E) Principal coordinate analysis showed similar gut microbial community structures among the full-time, early, and late antibiotic groups, whereas other groups showed distinct gut microbial profiles. (F) A tendency toward a positive correlation between number of tumors and number of OTUs was observed. (G, H) The relative abundance of Bacteroidales order and Lachnospiraceae family tended to be positively related to tumor burden. AOM, azoxymethane; DSS, dextran sodium sulfate; ABX, antibiotics, OTU, operational taxonomic unit. * *p*<0.05.

Fig 6 shows the changes in gut microbiota associated with antibiotic administration. Chao 1 richness was reduced in all antibiotic treatment groups, especially in the early and late antibiotic groups. Rarefaction curves also revealed significantly decreased number of observed OTUs in the full-time, early, and late antibiotic groups. However, reduction in gut microbial diversity was less prominent in the pretreatment antibiotic group. Quantitative 16S rRNA PCR on fecal samples showed that the overall bacterial load in the antibiotic-treated groups was not significantly reduced compared to that of the control and AOM/DSS groups.

At the phylum level, the relative abundance of Proteobacteria was significantly higher in the full-time, early, and late antibiotic groups than the other groups. Principal coordinate analysis showed similar gut microbial community structures among the full-time, early, and late antibiotic groups. In contrast, distinct intestinal microbial profiles were present between the control, AOM/DSS, and pretreatment antibiotic groups. In addition, there was a tendency toward a positive correlation between number of tumors and number of OTUs. The relative abundance of the Bacteroidales order and Lachnospiraceae family tended to be positively related to tumor burden.

These findings suggest that the gut microbial diversity and community structure altered by the antibiotic treatment may be involved in colon tumorigenesis.

## Discussion

In this study, we demonstrated that manipulation of the gut microbiota using antibiotics attenuates colon tumorigenesis in the AOM/DSS-induced murine CAC model. In addition, there were significant differences in development of CAC according to timing of gut microbial change through antibiotic administration. Colon tumorigenesis was most markedly suppressed when antibiotics were administered before onset of inflammation induced by DSS and throughout the whole period of inflammation. In contrast, colon tumorigenesis was not suppressed when antibiotics were administered only before DSS administration.

Previous studies have shown that inflammatory cells and related mediators, including IL-6, TNF-α, and IL-23, enhance DNA damage in intestinal epithelial cells to form a carcinogenic microenvironment during development of CAC [11–13]. Persistent inflammatory cytokines also promote barrier dysfunction, making intestinal epithelial cells vulnerable to bacterial invasion and leading to further inflammatory responses [5]. In addition, inflammation modifies gut microbial composition and can induce expansion of microbes with genotoxic potential to promote tumorigenesis [14]. Altogether, these results suggest that colon tumorigenesis is a complex process involving inflammation, microbes, and cancer cell interactions [6]. Attempts to suppress tumorigenesis have been made by manipulating gut microbiota, given that microorganisms are responsible for one axis of tumorigenesis, and the results have shown that manipulation of gut microbiota using antibiotics suppressed colon tumorigenesis in the AOM/DSS model [8, 9]. Although these results demonstrate the possible role of gut microbial change in suppressing tumorigenesis, it is currently unclear which stage of the inflammation-dysplasia-carcinoma sequence is most impacted by changes in gut microbial composition. Our results showed that colon tumorigenesis was not suppressed in the group that received only antibiotics prior to induction of inflammation, whereas tumorigenesis was suppressed in the group that received full-time antibiotics and the group that received antibiotics after induction of inflammation. These results suggest that maintaining gut microbial changes continuously during inflammation is crucial for suppressing colon tumorigenesis, because the altered structure of the gut microbial community caused by antibiotic administration may be restored immediately after antibiotic treatment [15].

CAC develops through a sequence of no dysplasia-indefinite dysplasia-low grade dysplasia-high grade dysplasia-carcinoma [11]. In other words, colitis-related neoplastic lesions basically arise within inflamed areas of the colon [11, 16]. However, tumor progression may skip one or more steps of the inflammation-dysplasia-carcinoma sequence and can occur in IBD patients without dysplasia [16–18]. In addition, although the presence of dysplasia is the most reliable marker for identifying developing carcinoma in patients with IBD [16], a number of dysplasias do not progress or may regress [19, 20]. A previous study demonstrated that medical therapy with aminosalicylates was associated with decreased risk of neoplasia progression in UC patients with indefinite dysplasia [21]. Therefore, controlling gut inflammation is crucial to suppress tumorigenesis. Our results demonstrated a robust effect of tumor suppression in the full-time antibiotic group, but the effect was weaker in the group that received antibiotics in the first or second round of DSS in a stepwise fashion. These results suggest that gut microbial changes should be maintained throughout the inflammation-dysplasia-carcinoma sequence to effectively suppress tumorigenesis. Meanwhile, the group that received antibiotics with the second round of DSS showed moderate decreases in tumor burden, suggesting that gut microbial changes have some degree of tumor suppression effect even after the process of dysplasia has begun. Our results may provide a better understanding of the impact of gut microbial manipulation with antibiotics on the sequential stages of tumorigenesis in the AOM/DSS-induced CAC model.

Several specific microbes, including *Streptococcus gallolyticus*, *Enterococcus faecalis*, colibactin-producing *Escherichia coli*, enterotoxigenic *Bacteroides fragilis*, and *Fusobacterium nucleatum*, are involved in development of CRC through modulation of the tumor immune environment and promotion of DNA damage [6]. However, individual bacteria as well as the entire intestinal microbial community can act as potential promoters of carcinogenesis [6, 8, 9, 22]. Inflammation and microbiota contribute to tumorigenesis through complex intertwining relationships, and there is evidence that multiple microbes can work together to promote tumorigenesis [6, 8, 23, 24]. In our principal coordinate analysis, distinct microbial community structures were seen between groups that tumorigenesis was inhibited and was not inhibited. These results suggest that changes in gut microbial community structure rather than a single individual microorganism play an important role in tumorigenesis. Meanwhile, the relative abundance of the Bacteroidales order and Lachnospiraceae family was positively correlated with number of tumors. However, given the low numbers of observed OTUs in the antibiotic-treated groups, it cannot be concluded that the Bacteroidales order and Lachnospiraceae family play a crucial role in development of CAC.

In our results, the number of OTUs was significantly reduced in the groups in which tumorigenesis was suppressed, resulting in an overall positive correlation between number of OTUs and tumor burden. Additionally, Chao 1 index, one of the alpha diversity indices, was reduced in the antibiotic treated groups than in the control and AOM/DSS groups. These results suggest that gut microbial diversity may play an important role in tumorigenesis. However, caution is needed in interpreting results, because the total amount of microorganisms may also be reduced by the antibiotic treatment. Increased bacterial load in colon tissue is known to contribute to intestinal carcinogenesis [25]. However, the reduced number of OTUs does not necessarily mean that the total amount of microbiota was reduced because there is no simple correlation between the number of OTUs and the absolute microbial load. In addition, our quantitative 16S rRNA PCR on fecal samples demonstrated that antibiotic treatment did not significantly reduce the total microbial amount. Previous studies also have shown that antibiotics modify the composition and diversity of gut microbiota but have no significant effect on the total amount of gut microbiota [8, 26]. Therefore, although it is unclear which of the total amount or diversity of gut microbiota had a greater effect on colon tumorigenesis, our data suggest that changes in gut microbial diversity and community structure may affect tumorigenesis more significantly than the overall gut microbial load.

In conclusion, we found that manipulation of gut microbiota using antibiotics can attenuate colon tumorigenesis in the AOM/DSS model. In particular, gut microbial changes should be maintained throughout the entire period of inflammation to effectively suppress colon tumorigenesis. Manipulation of gut microbiota using antibiotics may be considered as a potential therapeutic option for CAC.

## Supporting information

S1 Table Scoring system for determining humane endpoint. The humane endpoint was determined to have a total score of the sum of each item ≥8, or the highest points in two or more of the individual items.
(PDF)
