# Supplementary material for: The impact of gut microbiota manipulation with antibiotics on colon tumorigenesis in a murine model
Source: PLoS One. 2019 Dec 20;14(12):e0226907. doi: 10.1371/journal.pone.0226907 (PMC6924659; doi:10.1371/journal.pone.0226907)
Supplement: S1 Table — The humane endpoint was determined to have a total score of the sum of each item ≥8, or the highest points in two or more of the individual items. (PDF) [file pone.0226907.s001.pdf]

S1 Table. Scoring system for determining humane endpoint

Score	Body weight	Hair coat	Eye and nose	Activity	Posture
0	Normal	Normal	Normal	Normal	Normal
1	10% reduction	Coarse hair	Half-closed eyes	Reduced activity<br>Reduced response to stimuli	Bent posture
2	20% reduction	Hair loss<br>Inadequate grooming	Closed eyes<br>Periorbital swelling<br>Porphyrin staining	No response to stimuli	Unable to hold the head up
3	30% reduction				Lying on the floor

The humane endpoint was determined to have a total score of the sum of each item ≥8, or the highest points in two or more of the individual items.
